# Evaluation of the Etest and disk diffusion method for detection of the activity of ceftazidime-avibactam against *Enterobacterales* and *Pseudomonas aeruginosa* in China

**DOI:** 10.1186/s12866-020-01870-z

**Published:** 2020-06-29

**Authors:** Qi Wang, Feifei Zhang, Zhanwei Wang, Hongbin Chen, Xiaojuan Wang, Yawei Zhang, Shuguang Li, Hui Wang

**Affiliations:** Department of Clinical Laboratory, Peking University People’s Hospital, Beijing, 100044 China

**Keywords:** Ceftazidime-avibactam, Etest, Broth microdilution, Disk diffusion

## Abstract

**Background:**

Ceftazidime-avibactam was approved in China in 2019 for treating complicated intra-abdominal infections, hospital-acquired pneumonia, ventilator-associated pneumonia, and infections caused by *Enterobacterales* and *Pseudomonas aeruginosa* for which treatment options are limited. However, no currently available commercial systems have been approved for antimicrobial susceptibility testing of ceftazidime-avibactam in China. Here, we evaluated the Etest and disk diffusion method for detecting the activity of ceftazidime-avibactam against *Enterobacterales* and *P. aeruginosa* in China.

**Results:**

In total, 194 *Enterobacterales* and 77 *P. aeruginosa* isolates, which were divided into a random selection group (140 *Enterobacterales* and 46 *P. aeruginosa* isolates) and stock group (54 *Enterobacterales* and 31 *P. aeruginosa* isolates), were assessed by the Etest, disk diffusion and broth microdilution methods. Minimum inhibitory concentrations and zone diameters were interpreted according to the CLSI supplement M100 30th edition. For all 271 tested isolates, no very major errors were found by using Etest, whereas the overall major error rate was 2.0% (4/203). The overall categorical agreement rates of Etest for *Enterobacterales* and *P. aeruginosa* were 99.5% (193/194) and 96.1% (74/77), respectively, and the essential agreement rates were 95.9% (186/194) and 94.8% (73/77), respectively. The disk diffusion method showed that the very major error and major error rates were 1.5% (3/204) and 2.5% (5/203), respectively. Overall categorical agreement rates values of the disk diffusion method for *Enterobacterales* and *P. aeruginosa* were 98.5% (191/194) and 93.5% (72/77) compared with broth microdilution, respectively.

**Conclusions:**

For *Enterobacterales* and *P. aeruginosa*, both the Etest and disk diffusion method showed acceptable performance as alternatives to the standard broth microdilution method for clinical treatment interpretation. Application of the disk diffusion method in *Enterobacterales* was slightly better than that in *P. aeruginosa*.

## Background

Gram-negative bacilli, particularly carbapenem-resistant *Enterobacterales* (CRE) and *Pseudomonas aeruginosa*, exhibit major antimicrobial resistance worldwide, including in European countries, the United States (USA), and China [1–3]. The approval of ceftazidime-avibactam for clinical use in Europe and the USA has provided new treatment options for CRE-infected patients, particularly those with serine-carbapenemase resistance mechanisms [4, 5]. Ceftazidime-avibactam was approved in China in 2019 for treating complicated intra-abdominal infections, hospital-acquired pneumonia, ventilator-associated pneumonia, and infections caused by *Enterobacterales* and *P. aeruginosa* without enough effective treatment options. In China, there is currently no automated system for antimicrobial susceptibility testing of ceftazidime-avibactam. Ceftazidime-avibactam susceptibility tests are urgently needed to facilitate appropriate targeted treatment in patients with infections caused by multidrug-resistant *Enterobacterales* and *P. aeruginosa*. Although ceftazidime-avibactam has excellent in vitro activity against carbapenem-resistant *Klebsiella pneumoniae* (CRKP) and *P. aeruginosa* isolates, a few resistant isolates remain during treatment or nosocomial infection transmission [6–8]. Therefore, the susceptibility test results for ceftazidime-avibactam are even more critical.

In most laboratories in China, performing the standard broth microdilution (BMD) method is challenging. Therefore, alternative methods are needed to reliably determine ceftazidime-avibactam susceptibility. In this study, we evaluated two antimicrobial susceptibility testing methods for ceftazidime-avibactam, i.e., the Etest and disk diffusion method with the reference BMD, to evaluate whether these simple methods can be utilized for testing ceftazidime-avibactam in the clinical setting.

## Results

### Etest versus the BMD

**Fig. 1:**
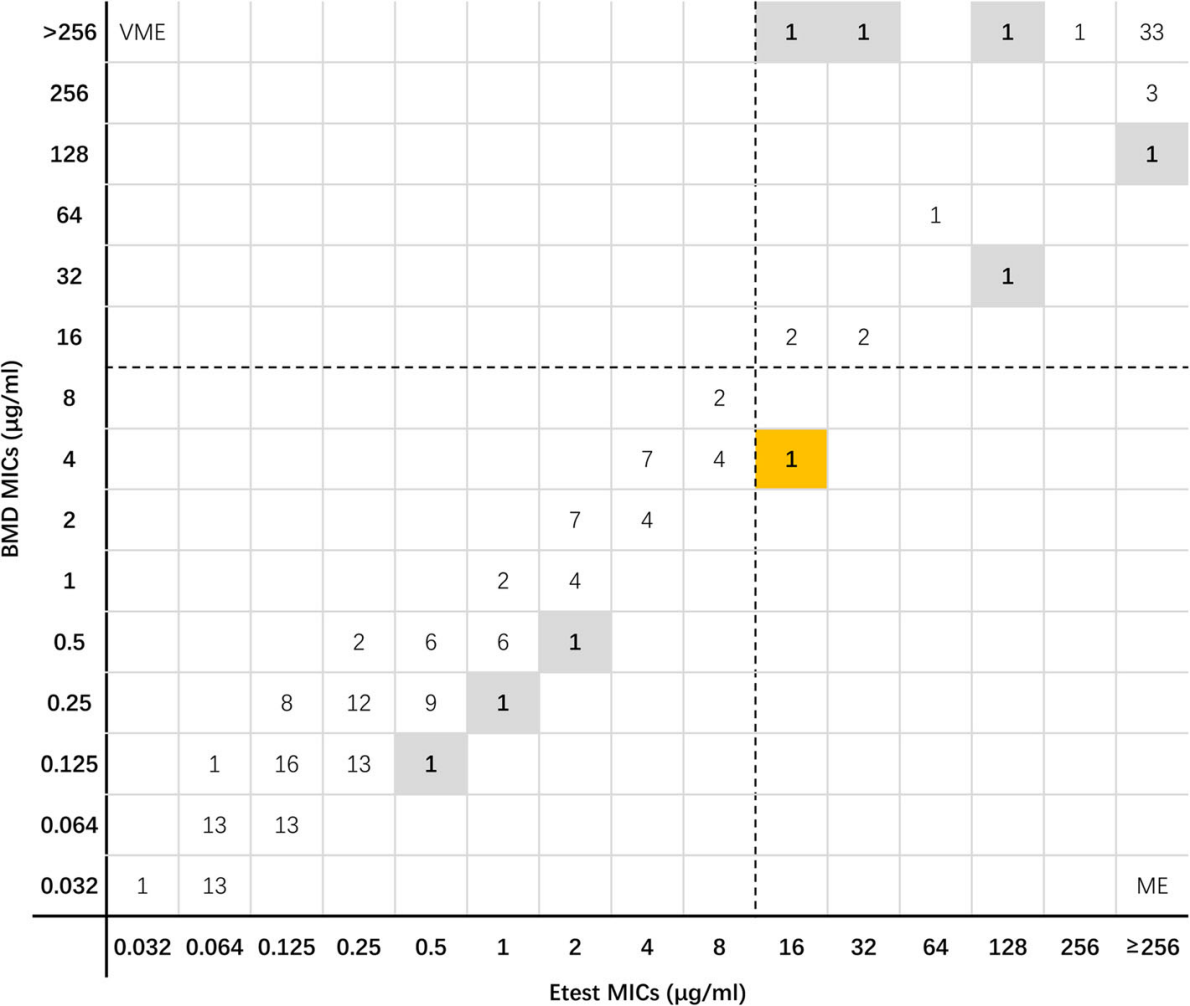
Scatter plot of ceftazidime-avibactam Etest MICs versus BMD MICs against *Enterobacterales*. Dotted lines represent the susceptibility breakpoint for ceftazidime-avibactam. VME: very major error (false susceptible); ME: major error (false resistant). The gray background indicates that the MIC of the Etest did not satisfy the essential agreement compared with the MIC of the BMD; the yellow background indicates that a major error occurred in the MIC of the Etest compared with the MIC of the BMD

According to the BMD method, 24.2% (47/194) of *Enterobacterales* and 28.4% (21/74) of *P. aeruginosa* were resistant to ceftazidime-avibactam. For all 194 *Enterobacterales* isolates, no very major error (VME) were found using the Etest. One isolate in the stock group was classified as resistant by Etest but susceptible by BMD. The major error (ME) rate was 0.7% (1/147) in *Enterobacterales*. As shown in Table 1, the overall categorical agreement (CA) rate was 99.5%, and the overall essential agreement (EA) rate was 95.9%. The CA rate of the stock group was 98.1%, and that in the random selection group was 97.9%. When comparing Etest results with the BMD results, the MICs of nine isolates exceeded the two-fold dilution. As shown in Fig. 1, the Etest minimum inhibitory concentrations (MICs) of 102 (52.5%) isolates were consistent with the BMD MICs. The Etest MICs of 71 (36.6%) isolates were one-fold dilution higher than the BMD MICs. Only 12 (6.2%) isolates showed Etest MICs that were one-fold dilution lower than the BMD MICs.

**Table 1:** Evaluation of essential and categorical agreement between the BMD method and Etest or disk diffusion method for analysis of ceftazidime-avibactam antimicrobial susceptibility

Organism	No. of isolates tested	No. of resistant isolates by BMD	E-test				Disk diffusion		
			No. (%) of CA	No. (%) of EA	No. (%) of VME	No. (%) of ME	No. (%) of CA	No. (%) of VME	No. (%) of ME
***Enterobacterales***									
Random selection group	140	7	140 (100)	137 (97.9)	0 (0)	0 (0)	140 (100)	0 (0)	0 (0)
Stock group	54	40	53 (98.1)	49 (90.7)	0 (0)	1 (7.1)	51 (94.4)	2 (5.0)	1 (7.1)
Total in *Enterobacterales*	194	47	193 (99.5)	186 (95.9)	0 (0)	1 (0.7)	191 (98.5)	2 (4.3)	1 (0.7)
***P. aeruginosa***									
Random selection group	46	6	46 (100)	45 (97.8)	0 (0)	0 (0)	43 (93.5)	0 (0)	3 (7.5)
Stock group	31	15	28 (90.3)	28 (90.3)	0 (0)	3 (18.7)	29 (93.5)	1 (6.7)	1 (6.3)
Total in *P. aeruginosa*	77	21	74 (96.1)	73 (94.8)	0 (0)	3 (5.4)	72 (93.5)	1 (4.8)	4 (7.1)
**Total in all tested isolates**	271	68	267 (98.5)	259 (95.6)	0 (0)	4 (2.0)	263 (97.0)	3 (1.5)	5 (2.5)

*EA* essential agreement, *CA* categorical agreement, *VME* very major error (false susceptible), *ME* major error (false resistant)

**Fig. 2:**
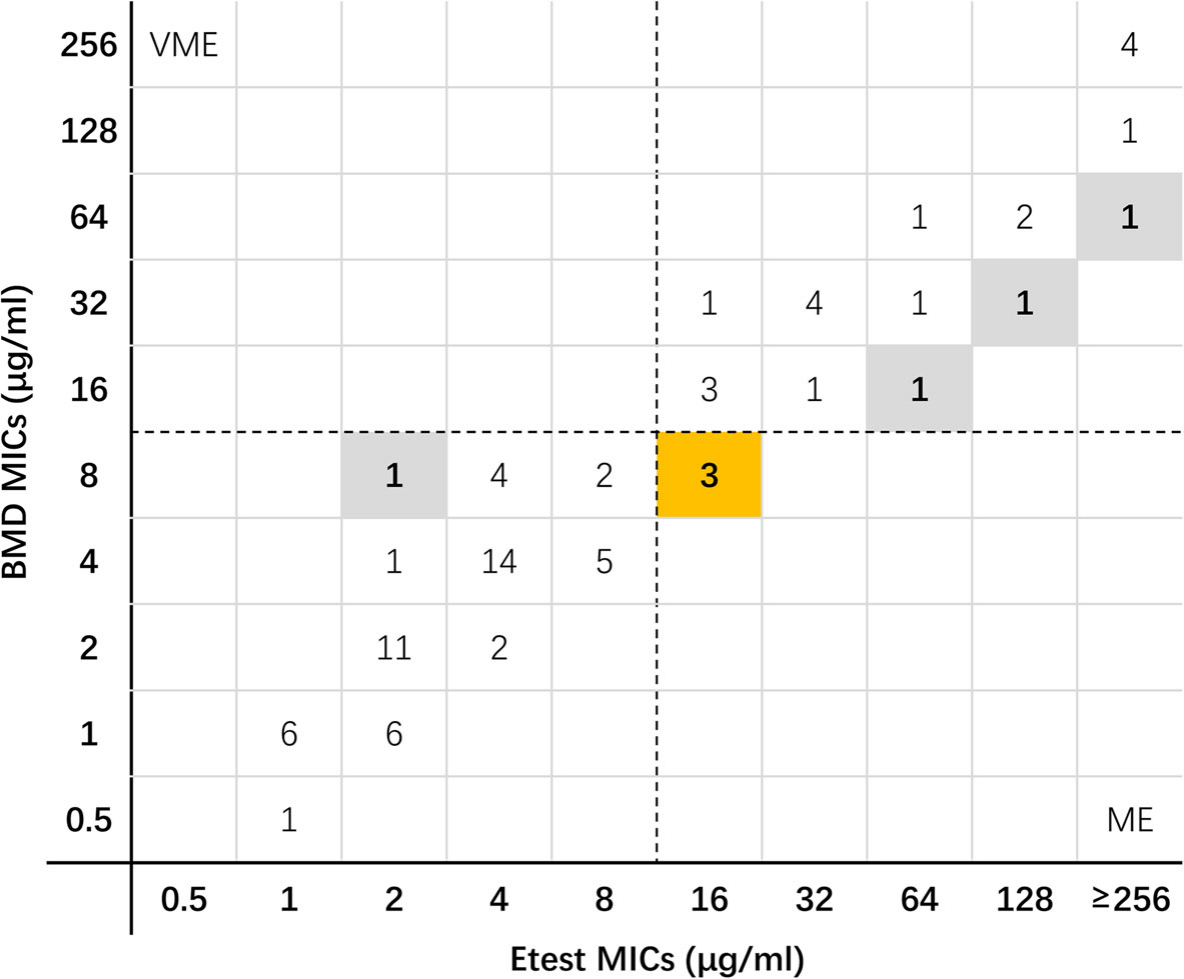
Scatter plot of ceftazidime-avibactam Etest MICs versus BMD MICs against *Pseudomonas aeruginosa*. Dotted lines represent the susceptibility breakpoint for ceftazidime-avibactam. VME: very major error (false susceptible); ME: major error (false resistant). The gray background indicates that the MIC of the Etest did not satisfy the essential agreement compared with the MIC of the BMD; the yellow background indicates that three major errors occurred in the MIC of the Etest compared with the MIC of the BMD

For 77 *P. aeruginosa* isolates, no VME were found using the Etest method. Three isolates in the stock group were classified as resistant by Etest but susceptible by BMD. As shown in Table 1, the overall CA and EA rates were 96.1% and 94.8%, respectively. The CA rates in the stock and random selection groups were 90.3% and 100%, respectively. As shown in Fig. 2, the Etest MICs of 45 (58.4%) isolates were consistent with those obtained by the BMD. The Etest MICs of 22 (28.6%) isolates were one-fold dilution higher than those obtained by the BMD, whereas those of six (7.8%) isolates were one-fold dilution lower than the MICs obtained by the BMD. For one strain, the Etest MIC was two-fold dilutions higher than that obtained by the BMD. Three MEs appeared in the stock group when the Etest method was used.

### Disk diffusion method versus BMD

**Fig. 3:**
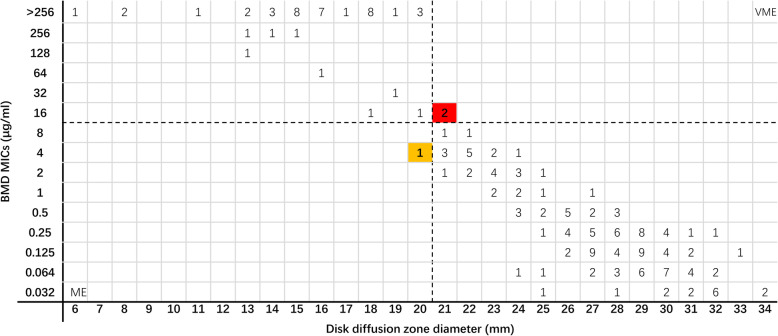
Scatter plot of ceftazidime-avibactam zone diameters versus BMD MICs against *Enterobacterales*. Dotted lines represent the susceptibility breakpoint for ceftazidime-avibactam. VME: very major error (false susceptible); ME: major error (false resistant). The yellow background indicates that a major error occurred for the disk diffusion method compared with the BMD. The red background indicates that two very major errors occurred when the disk diffusion method was compared with the BMD

A comparison of the disk diffusion method and BMD results for 194 *Enterobacterales* isolates are shown in Fig. 3. The overall CA rate in the 194 *Enterobacterales* isolates was 98.5%. Two VMEs and 1 ME were found using the disk diffusion method in the stock group; all were carbapenem-resistant *K. pneumoniae* carbapenemase (KPC)-producing isolates. No VME or ME were found using the disk diffusion method in the random selection group. There were 22 isolates of *Enterobacterales* with zone diameters between 19 and 22 mm. Forty-one of the 47 resistant isolates obtained by BMD showed zone diameters in the range of 13–20 mm.

**Fig. 4:**
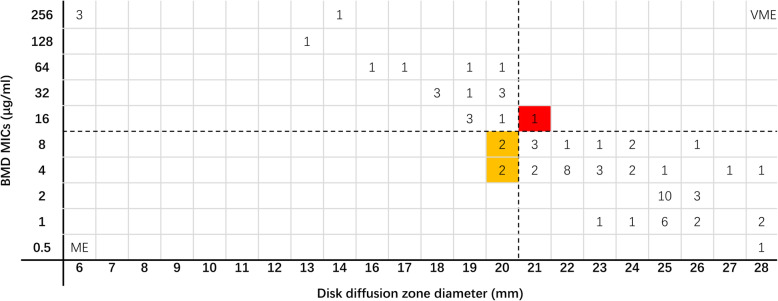
Scatter plot of ceftazidime-avibactam zone diameters versus BMD MICs against *Pseudomonas aeruginosa.* Dotted lines represent the susceptibility breakpoint for ceftazidime-avibactam. VME: very major error (false susceptible); ME: major error (false resistant). The yellow background indicates that four major errors occurred for the disk diffusion method compared with the BMD. The red background indicates that a very major error occurred when the disk diffusion method was compared with the BMD

Comparison of the disk diffusion method and BMD for 77 *P. aeruginosa* isolates showed that the ME% was 7.1%. As shown in Table 1, the overall CA rate was 93.5%. Moreover, as shown in Fig. 4, when the zone diameter was 20 mm, the MICs obtained by the BMD method were 4, 8, 16, 32, and 64 μg/mL.

## Discussion

In the past decade, the incidence of CRE, particularly CRKP, has increased significantly in China. The latest China Antimicrobial Resistance Surveillance System data showed that the incidence of CRKP nationwide was as high as 10.1% (http://www.carss.cn/Report/Details?aId=648). However, few active antibacterial agents, such as tigecycline and colistin, are available to treat CRKP in the clinical setting, resulting in high mortality worldwide [9, 10]. Previous molecular epidemiological data revealed that more than 70% of CRE isolated in China from 2012 to 2016 produce KPC-type carbapenemases [11]. Ceftazidime-avibactam, a drug with potent antibacterial activity against serine-carbapenemase, was approved for use in China in 2019. Recent retrospective studies showed that ceftazidime-avibactam treatment of CRKP and *P. aeruginosa* infection in patients who have undergone solid organ transplantation improves clinical success rates [12, 13]. Despite these promising findings, ceftazidime-avibactam antimicrobial susceptibility test results are essential for the clinical use of this treatment. Additionally, commercial automated systems in the clinical microbiology laboratory cannot be used for antimicrobial susceptibility testing of ceftazidime-avibactam in China.

This is the first study to compare the Etest, disk diffusion method, and BMD to detect ceftazidime-avibactam susceptibility in China. We use BMD as a standard clinical method for comparison with the other methods. Compared with the standard BMD method, no VME were found using the Etest method. The results of Etest MICs and BMD MICs were reasonably well correlated for both *Enterobacterales* and *P. aeruginosa*. The overall EA% of 271 tested isolates was 95.6%. Compared with the BMD method, the Etest method exhibited an excellent linear correlation, supporting the use of this approach as an alternative to the standard clinical method without considering economic costs. The Etest results were similar to those of previous research findings [14]. A study of the ceftazidime-avibactam Etest in Germany reported excellent results in terms of EA and CA for susceptibility testing of *Enterobacterales* and *P. aeruginosa* [15]. A total of 140 *Enterobacterales* and 60 *P. aeruginosa* were examined; the total EA% and CA% were 99.0% and 99.5%, respectively.

The disk diffusion method is easy to implement in the clinical setting from an economic perspective. The CA% values of the ceftazidime-avibactam disk diffusion method against *Enterobacterales* and *P. aeruginosa* were 98.5% and 93.5%, respectively. However, a previous study by Shields et al. showed that 28% of ceftazidime-avibactam susceptible CRE isolates were classified as resistant by disk diffusion. The ME% is significantly higher than that obtained in our study [14]. This difference in data may be related to differences in the disk manufacturer. Indeed, our results showed that application of the disk diffusion method was more appropriate for *Enterobacterales* than for *P. aeruginosa*. Notably, however, the disk diffusion method did not exhibit an excellent linear correlation with BMD. Moreover, for both *Enterobacterales* or *P. aeruginosa* against ceftazidime-avibactam, the disk diffusion method tends to show falsely susceptible results. As the sample size in our study was small, the number of VME was low. Therefore, clinical verification of larger sample sizes is needed.

Based on our results, when the zone diameters of ceftazidime-avibactam against *Enterobacterales* and *P. aeruginosa* were 20–21 mm, BMD testing should be performed to avoid false-susceptible or false-resistant results. Our results were consistent with those of other studies [16, 17]. This should also be considered when users refer to the new version of CLSI M100, which suggests using the disk diffusion method for *Enterobacterales* [18]. The CLSI recommends that MICs should be determined if the zone for Enterobacterales isolates is 20–22 mm. CLSI does not have a similar comment for *P. aeruginosa*.

## Conclusions

In conclusion, for *Enterobacterales* and *P. aeruginosa*, ceftazidime-avibactam Etest and the disk diffusion method showed acceptable performance as alternatives to the BMD method for clinical treatment interpretation. Application of the disk diffusion method for *Enterobacterales* was slightly better than that for *P. aeruginosa*.

## Methods

### Bacterial groups

Isolates were divided into two groups, i.e., random selection group and stock group (Table S1). For the random selection group, we randomly selected 140 *Enterobacterales* and 46 *P. aeruginosa* isolates from clinical non-repeated isolates obtained from Peking University People’s Hospital. Among these isolates, 59.3% (83/140) of *Enterobacterales* and 56.5% (26/46) of *P. aeruginosa* isolates were defined as fresh clinical isolates obtained within 1 month prior to testing (November 2019 to March 2020). Based on the collection of fresh clinical isolates, we conducted three batches of antimicrobial susceptibility tests, each in parallel with three methods (BMD, Etest, and disk diffusion method). The first test was conducted in December 2019, and the testing isolates were isolated within 1 month before the test date. The second and third tests were conducted in January 2020 and March 2020, respectively, and the test isolates were isolated within 1 month before the test date. The remaining isolates in the random selection group were obtained from the strain repository of Peking University People’s Hospital from January 2018 to October 2019. The 140 isolates of *Enterobacterales* used for testing included 13 species, i.e., 25 *K. pneumoniae*, 19 *Escherichia coli*, 18 *Proteus mirabilis*, 17 *Enterobacter cloacae*, 16 *Serratia marcescens*, 15 *Citrobacter freundii*, 14 *K. oxytoca*, 4 *Proteus vulgaris*, 3 *Morganella morganii*, 3 *Providencia stuartii*, 2 *Providencia rettgeri*, 2 *K. aerogenes*, and 2 *Citrobacter koseri* isolates. For the stock group, we selected 54 isolates of *Enterobacterales* from 15 hospitals in the CRE China-Network from January 2015 to October 2019 and requested MICs of ceftazidime-avibactam of 2–>256 μg/mL. Among these isolates, six (11.1%) showed MICs for ceftazidime-avibactam of 8–16 μg/mL, and 15 isolates (27.8%) showed MICs of 4–32 μg/mL. These isolates with known MICs were mainly used to verify the accuracy of the value near the ceftazidime-avibactam breakpoint. The carbapenem-resistance genes present in these isolates were determined in previous studies [11]. The 54 isolates of *Enterobacterales* used in this study included 29 *K. pneumoniae* (18 with *bla*_KPC_ and 10 with *bla*_NDM_), 12 *E. coli* (2 with *bla*_KPC_ and 7 with *bla*_NDM_), 8 *E. cloacae* (1 with *bla*_KPC_, 5 with *bla*_NDM_, 1 with *bla*_IMP_, and 1 with *bla*_VIM_), 3 *K. oxytoca* (2 with *bla*_IMP_ and 1 with *bla*_NDM_), and 2 *Citrobacter freundii* (1 with *bla*_IMP_ and 1 with *bla*_NDM_).

We selected 31 isolates of *P. aeruginosa* from the 8 hospitals involved in the Chinese Antimicrobial Resistance Surveillance of Nosocomial infections 2018 project as stock group isolates. The MICs of ceftazidime-avibactam were 2–>256 μg/mL. Among these isolates, 12 (38.7%) had MICs for ceftazidime-avibactam between 8 and 16 μg/mL and 25 (80.6%) had MICs between 4 and 32 μg/mL. These *P. aeruginosa* isolates with known MICs are mainly used to verify the accuracy of the value near the ceftazidime-avibactam breakpoint.

All isolates were removed from a − 80 °C ultra-low temperature freezer and transferred to Columbia blood agar twice before antimicrobial susceptibility testing.

### Antimicrobial susceptibility testing

For the disk diffusion method, ceftazidime-avibactam disks were obtained from Oxoid (Hampshire, UK). The content of ceftazidime-avibactam in each disk was 30 μg/20 μg. Testing were performed precisely according to the recommendations of the CLSI [19].

For the Etest gradient diffusion method, ceftazidime-avibactam Etest strips were obtained from BioMérieux (Marcy l’Etoile, France). The tests were performed in strict accordance with the manufacturer’s instructions. The ceftazidime concentration gradient ranged from 0.016 to 256 μg/mL with avibactam at a constant concentration of 4 μg/mL. When the Etest MIC value was between the standard value and twice the standard value (0.016, 0.032, 0.064, 0.125, 0.25, 0.5, 1, 2, 4, 8, 16, 32, 64, 128, and 256), the high standard value was considered as the MIC.

The MH agar plates used for both the disk diffusion method and Etest gradient diffusion method for antimicrobial susceptibility testing were obtained from Oxoid.

The BMD was performed strictly following CLSI guidelines [18]. Ceftazidime and avibactam powder were obtained from MedChemExpress (Monmouth Junction, NJ, USA). The ceftazidime concentration ranged from the standard double dilution of 0.016–256 μg/mL. The concentration of avibactam was fixed at 4 μg/mL.

Quality controls were evaluated simultaneously in each batch of experiments. Colony counting was performed to monitor the inoculum density. *Escherichia coli* ATCC 25922, *K. pneumoniae* ATCC 700603, *E. coli* ATCC 35218, and *P. aeruginosa* ATCC 27853 were used as experimental quality control isolates. The tests were considered as valid only when the results for all quality control isolates were within the acceptable range.

The MICs and zone diameters of ceftazidime-avibactam for *Enterobacterales* and *P. aeruginosa* were interpreted according to the CLSI supplement M100 30th edition [18]. Briefly, MICs of ≤8/4 μg/mL or a zone diameter of ≥21 mm indicated that the strain was susceptible, whereas MICs of ≥16/4 μg/mL or a zone diameter of ≤20 mm indicated that the strain was resistant.

Essential agreement (EA) indicated that the difference between the MIC value measured by Etest and the BMD did not exceed one two-fold dilution. CA indicated that interpretive category results for the Etest method or disk diffusion method were the same as those for the reference BMD using CLSI breakpoints. VME indicated that the strain was susceptible according to the Etest or the disk diffusion method but resistant according to the BMD. ME indicated that the strain was susceptible by the BMD but resistant by Etest or the disk diffusion method.

## Supplementary information

**Additional file 1: Table S1.** Organisms and distribution of carbapenem resistance mechanisms in this study.

## Data Availability

All documents and additional data are available from the corresponding author upon reasonable request.

## Abbreviations

BMD: Broth microdilution
MIC: Minimum inhibitory concentration
VME: Very major error
CLSI: Clinical & Laboratory Standards Institute
ME: Major error
CA: Categorical agreement
EA: Essential agreement
CRKP: Carbapenem-resistant *Klebsiella pneumoniae*
CRE: Carbapenem-resistant *Enterobacterales*
*bla*_KPC_: Beta-lactamase gene, *Klebsiella pneumoniae* carbapenemase
*bla*_NDM_: Beta-lactamase gene, New Delhi metallo-β-lactamase
*bla*_IMP_: Beta-lactamase gene, imipenemase
ATCC: American Type Culture Collection
