# Supplementary material for: Evaluation of the Etest and disk diffusion method for detection of the activity of ceftazidime-avibactam against Enterobacterales and Pseudomonas aeruginosa in China
Source: BMC Microbiol. 2020 Jun 29;20:187. doi: 10.1186/s12866-020-01870-z (PMC7325266; doi:10.1186/s12866-020-01870-z)
Supplement: Supplementary file 1 — Additional file 1: Table S1. Organisms and distribution of carbapenem resistance mechanisms in this study. [file 12866_2020_1870_MOESM1_ESM.docx]

**Table S1. Organisms and the distribution of carbapenem resistance mechanisms in this study**

| **Organisms** | | **No. of strains tested** | **Carbapenemase gene (no.)** |
| --- | --- | --- | --- |
| *Enterobacterales* |  | 194 |  |
|  | Random selection Group | 140 |  |
|  | *Klebsiella pneumoniae* | 25 |  |
|  | *Escherichia coli* | 19 |  |
|  | *Proteus mirabilis* | 18 |  |
|  | *Enterobacter cloacae* | 17 |  |
|  | *Serratia marcescens* | 16 |  |
|  | *Citrobacter freundii* | 15 |  |
|  | *Klebsiella oxytoca* | 14 |  |
|  | *Proteus vulgaris* | 4 |  |
|  | *Providencia stuartii* | 3 |  |
|  | *Morganella morganii* | 3 |  |
|  | *Providencia rettgeri* | 2 |  |
|  | *Klebsiella aerogenes* | 2 |  |
|  | *Citrobacter koseri* | 2 |  |
|  | Stock Group (CRE) | 54 |  |
|  | *Klebsiella pneumoniae* | 29 | *bla*_KPC_ (18); *bla*_NDM_ (10) |
|  | *Escherichia coli* | 12 | *bla*_KPC_ (2); *bla*_NDM_ (7) |
|  | *Enterobacter cloacae* | 8 | *bla*_KPC_ (1); *bla*_NDM_ (5); *bla*_IMP_ (1); *bla*_VIM_ (1) |
|  | *Klebsiella oxytoca* | 3 | *bla*_NDM_ (2); *bla*_IMP_ (1) |
|  | *Citrobacter freundii* | 2 | *bla*_NDM_ (1); *bla*_IMP_ (1) |
| *Pseudomonas aeruginosa* |  | 77 |  |
|  | Random selection Group | 46 |  |
|  | Stock Group | 31 |  |
